# Zbed3 promotes proliferation and invasion of lung cancer partly through regulating the function of Axin‐Gsk3β complex

**DOI:** 10.1111/jcmm.14001

**Published:** 2018-11-12

**Authors:** Xiuying Shi, Yang Zhao, Chuifeng Fan

**Affiliations:** ^1^ Department of Pathology College of Basic Medical Sciences of China Medical University Shenyang China; ^2^ Department of Hepatobiliary and Spleenary Surgery The Affiliated Shengjing Hospital China Medical University Shenyang China

**Keywords:** β‐catenin, Axin, NSCLC, Zbed3

## Abstract

Our previous work showed that Zbed3 is overexpressed in nonsmall cell lung cancer and that down‐regulation of Zbed3 inhibited β‐catenin expression and cancer cell proliferation and invasiveness. Here, we investigated Zbed3's ability to promote lung cancer cell proliferation and invasion and the involvement of the Axin/TPC/glycogen synthase kinase 3β (Gsk‐3β) complex to the response. Coimmunoprecipitation assays showed that wild‐type Zbed3 bound to Axin but a Zbed3 mutant lacking the Axin binding site did not. In A549 and H1299 lung cancer cells, Zbed3 overexpression promoted cancer cell proliferation and invasiveness, as well as Wnt signalling and expression of downstream mediators, including β‐catenin, cyclin D1 and MMP7 (*P* < 0.05). In contrast, the Zbed3 mutant failed to enhance β‐catenin expression (*P* > 0.05), and its ability to promote cancer cell proliferation and invasiveness was much less than wild‐type Zbed3 (*P* < 0.05). The ability of Zbed3 to increase β‐catenin levels was abolished by Axin knockdown in A549 cells (*P* > 0.05). Similarly, treating the cells with a GSK‐3β inhibitor abolished Zbed3's ability to increase β‐catenin levels and Wnt signalling. These results indicate that Zbed3 enhances lung cancer cell proliferation and invasiveness at least in part by inhibiting Axin/adenomatous polyposis coli/GSK‐3β‐mediated negative regulation of β‐catenin levels.

## 1 INTRODUCTION

Abnormal Wnt signalling contributes to the development and progression of many malignancies.^1^, ^2^, ^3^, ^4^ β‐catenin is a key mediator of Wnt signalling that is degraded via a complex composed of Axin, adenomatous polyposis coli (APC), and serin/threonine glycogen synthase kinase 3β (GSK‐3β) when Wnt signalling is not activated.^5^, ^6^ When activated, Wnt signalling activates Disheveled, which in turn inhibits phosphorylation of β‐catenin by the Axin/APC/GSK‐3β complex and prevents its degradation via the ubiquitin proteasome. β‐catenin is accumulated in cytoplasm and then translocated to the nucleus, where it binds to and activates transcription factor T‐cell factor/Lymphoid, enhancer, factor, thereby regulating expression of target genes.^5^, ^6^

Zbed3 belongs to the family of BED‐zinc finger proteins. At present, it is predicted that there are about 2000 BED‐zinc finger proteins in the creature, but most of them have not been verified at the protein level, and the related research is still in an initial stage. We previously observed that Zbed3 is overexpressed in nonsmall cell lung cancer (NSCLC) and acts as a positive regulator of β‐catenin to promote NSCLC malignancy.^7^ Moreover, abnormal Axin (Axin1) expression or function is seen in various cancers and is considered to be a potential therapeutic target.^8^, ^9^ In NIH3T3 cells, Zbed3 reportedly suppresses β‐catenin degradation by inhibiting the activity of the Axin/APC/GSK‐3β complex.^10^ Although the mechanism involved is not fully understood, it is known that Zbed3 binding to Axin is important for inhibition of β‐catenin phosphorylation by the complex.^10^ To better understand the molecular mechanism of action of Zbed3 in NSCLC, in the present study we investigated the effects of Zbed3 on β‐catenin expression and the proliferation and invasiveness of NSCLC cells.

## 2 MATERIALS AND METHODS

### 2.1 Cell culture and transfection

The A549 and NCI‐H1299 human lung carcinoma cell lines were cultured as described previously.^7^ Lipofectamine 2000 (Invitrogen, Carlsbad, CA, USA) was used for transfection according to the manufacturer's instructions. siRNA and cDNA clones included siRNA‐Axin (sc‐41450; Santa Cruz, CA, USA), negative control siRNA (sc‐37007; Santa Cruz), Zbed3 cDNA clones (RC203522; Origene, MD, USA), mutant Zbed3 cDNA clones (Takara, Dalian, China), and control empty plasmid (Takara). Mutant Zbed3 cDNA clones lack the 116‐122 amino acid sequence. Cells were treated with the GSK‐3β inhibitor TWS119 (CAS 601514‐19‐6; Santa Cruz) at 1 μmol/L for 48 hours.

### 2.2 Western blotting

Western blotting was performed as described previously.^7^ The primary antibodies used included anti‐Zbed3 (ab106383, 1:300; Abcam, HK), anti‐Axin (ab56475, 1:500; Abcam), anti‐β‐catenin (D10A8, 1:1000; Cell Signaling, USA), anti‐phospho‐β‐catenin (Ser33/37/Thr41; #9561, 1:1000; Cell Signaling), anti‐cyclin D1 (sc‐20044, 1:200; Santa Cruz), anti‐GSK‐3β (E‐11, sc‐377213, 1:500; Santa Cruz), and anti‐GADPH (ab8245, 1:1000; Abcam).

### 2.3 Immunoprecipitation

Immunoprecipitation assays were performed as described previously.^11^ Antibodies were added to 200 mg of proteins and gently rotated overnight at 4°C. Twenty‐five millilitres of protein A/G agarose beads (Beyotime, Jiangsu, China) was added to the immunocomplex and lightly rotated for 3 hours at 4°C. The mixture was then centrifuged at 1500 *g* for 5 minutes at 4°C, and the supernatant was removed. The precipitate was resuspended in a sample buffer after washing three times using ice‐cold radioimmunoprecipitation assay buffer, and then boiled for 5 minutes. The sample was centrifugated and the supernatant was collected to be examined by Western blotting.

### 2.4 Matrigel invasion

To investigate the invasiveness of cancer cells, Transwell assays were performed using polycarbonate membrane inserts (Corning, NY, USA) and Matrigel (BD Bioscience) as described previously.^7^ Twenty millilitres of Matrigel (1:3 dilution) was added to each insert. One hundred millilitres of cell suspension containing 3 × 10^5^ cells was added to the upper chamber after transfection for 48 hours. The cells were incubated for 48 hours and then the filters were stained with haematoxylin. Cells on the lower surface of the filter were counted in five random high‐magnification microscopes. Each experiment was performed at least three times independently.

### 2.5 Colony formation

To investigate lung cancer cell proliferation, colony formation assays were performed as described previously.^7^ One thousand cells were planted into 6‐cm cell culture dishes after transfection for 48 hours and incubated for 10 days. The cells were then stained with Giemsa stain. Colonies with more than 50 cells were counted under the microscope. Each experiment was performed at least three times independently.

### 2.6 MTT assays

The MTT assays were performed as described previously.^12^ Cells were transferred to 96‐well plates in a medium containing 10% FBS at 4000 cells per well 48 hours after transfection. The 3‐(4,5‐dimethylthiazol‐2‐yl)‐2,5‐diphenyl tetrazolium bromide (MTT) assay (Thermo Fisher, USA) was used to evaluate cell viability. Briefly, 50 μL of 5 mg/mL MTT solution was added to each well and the cells were incubated for 4 hours at 37°C. Then the medium was discarded. The resultant MTT formazan was solubilized in 150 μL of DMSO. Cell proliferation was examined each day from 48 hours after transfection (Day 0) for 5 days after the MTT treatment. The results were analysed spectrophotometrically at a test wavelength of 550 nm.

### 2.7 Dual‐luciferase assays

Dual‐luciferase assays were performed as described previously.^13^ Tumour cells were transfected with pGL3‐OT or pGL3‐OF reporter gene plasmids. pRL‐TK was used as control plasmid. The plasmids were incubated for 30 hours at 37°C. Dual‐Luciferase Assay System (Promega, Madison, WI, USA) was used to detect the expression of reporter genes according to the manufacturer's instruction. The experiment was performed at least three times.

### 2.8 Transplantation of tumour cells into nude mice

Four‐week‐old female BALB/c nude mice were (Charles River, Beijing, China) subcutaneously inoculated with 5 × 10^6^ tumour cells in the axilla. The mice were examined for tumour growth every week. The mice were killed 3 weeks after inoculation and autopsied for examination of tumour growth. The experiments were approved by the Institutional Animal Research Committee of China Medical University and according to experimental animal ethics guidelines issued by China Medical University.

### 2.9 Statistical analysis

Statistical analysis was performed using the SPSS statistical software package version 22.0 (SPSS Inc., Chicago, IL, USA). In vitro experiments were performed at least three times. Differences between groups were analysed using Student's *t* test for densitometry measurements, Matrigel invasion assays, colony formation assays, and MTT assays. Values of *P* < 0.05 were considered statistically significant.

## 3 RESULTS

### 3.1 Overexpression of Zbed3 in lung cancer cells up‐regulated Wnt signalling molecules and cancer cell proliferation and invasiveness

**Figure 1:**
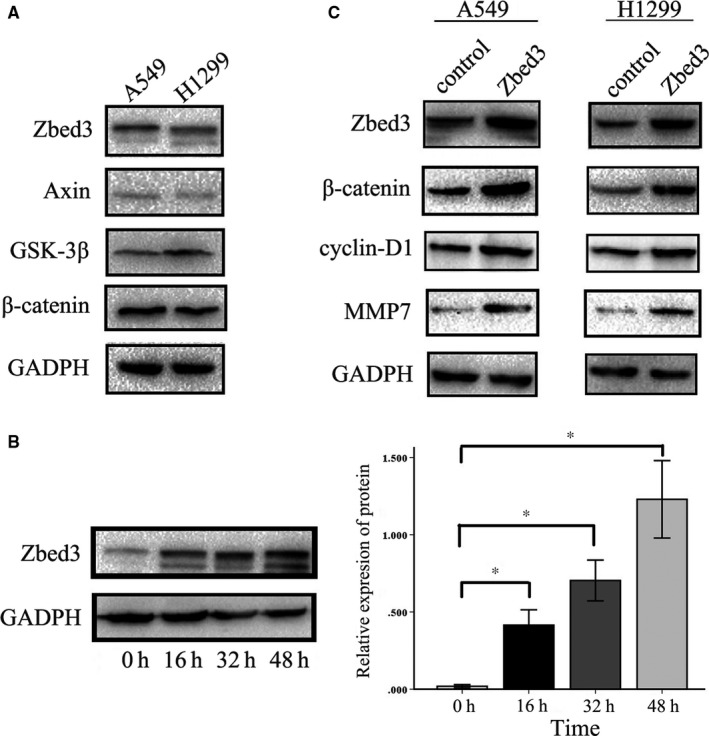
Overexpression of Zbed3 increases levels of Wnt signalling molecules in lung cancer cells. Western blots show Wnt signalling molecules, including Axin, GSK‐3β, and β‐catenin, are expressed in both A549 and NCI‐H1299 cells (A). Zbed3 expression is up‐regulated significantly in A549 cells 48 h after transfection (B). Zbed3 overexpression up‐regulates expression of β‐catenin and its downstream mediators, including cyclin‐D1 and MMP7, in A549 and NCI‐H1299 cells (C). The experiments were repeated three times (*P < 0.05)

**Figure 2:**
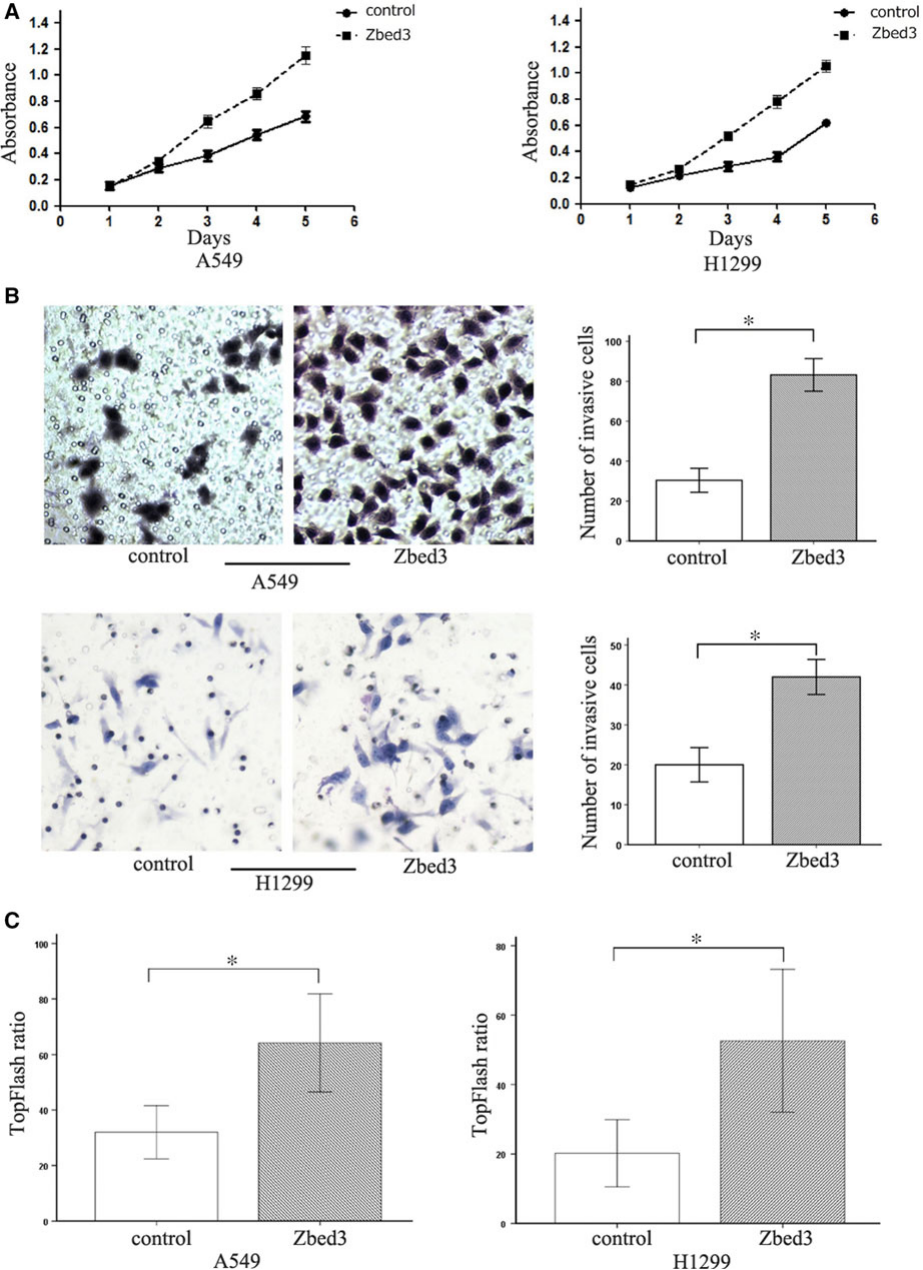
Overexpression of Zbed3 increases A549 and NCI‐H1299 lung cancer cell proliferation and invasiveness. MTT assays show that Zbed3 overexpression promotes cell proliferation (A). Transwell assays show that Zbed3 overexpression enhanced the invasiveness of the cells (B). Dual‐luciferase assays show that Zbed3 overexpression increases activity in the canonical Wnt signalling pathway (C). The experiments were repeated three times (*P < 0.05)

We first assessed expression of several Zbed3 and Wnt signalling molecules, including Axin, GSK‐3β, and β‐catenin in A549 and NCI‐H1299 lung cancer cells. Western blotting showed that all of these molecules were expressed in both A549 and NCI‐H1299 cells (Figure 1A). Transient transfection of the cells with a plasmid vector harbouring Zbed3 gene led to overexpression of the protein 48 hours after transfection (Figure 1B). Overexpression of Zbed3 significantly up‐regulated expression of β‐catenin and its downstream mediators, including cyclin D1, MMP7, and MMP7 (Figure 1C). Moreover, MTT assays showed that Zbed3 overexpression significantly enhanced cancer cell proliferation (Figure 2A), and Transwell assays showed it also enhanced the invasiveness of cancer cells (Figure 2B). In addition, dual‐luciferase assays showed that Zbed3 overexpression significantly increased activity in the canonical Wnt signalling pathway in these cells (Figure 2C).

### 3.2 Zbed3 mutation disrupting the Axin binding site abolished its effect on β‐catenin and p120ctn‐1 expression in lung cancer cells

**Figure 3:**
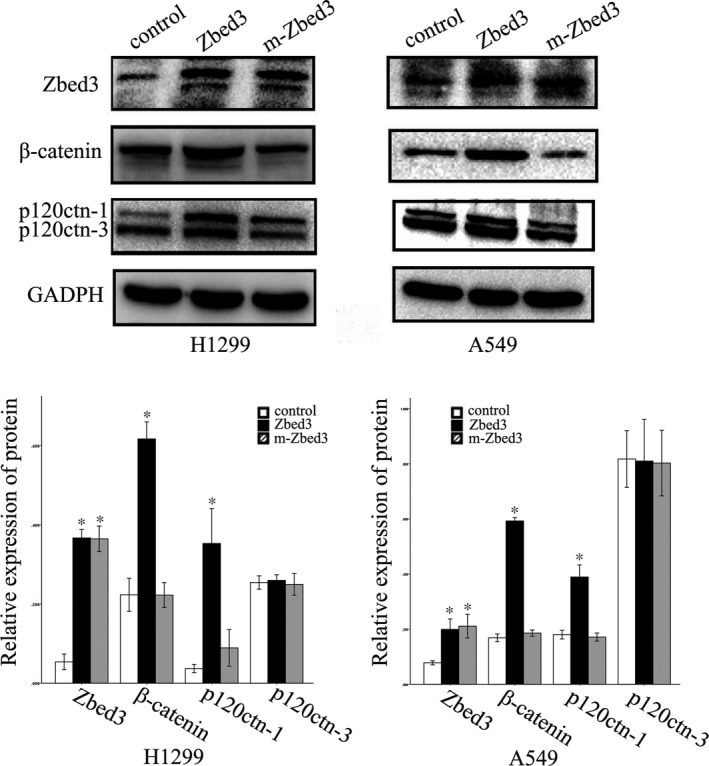
A Zbed3 mutant lacking an Axin binding site does not increase β‐catenin expression in NCI‐H1299 and A549 lung cancer cells. Overexpression of wild‐type Zbed3 increased β‐catenin and p120ctn‐1 expression. Transfection of the Zbed3 mutant failed to increase β‐catenin expression. The ability of regulating p120ctn‐1 was also significantly inhibited when transfected with the Zbed3 mutant compared to the wild‐type Zbed3. The experiments were repeated three times (*P < 0.05)

**Figure 4:**
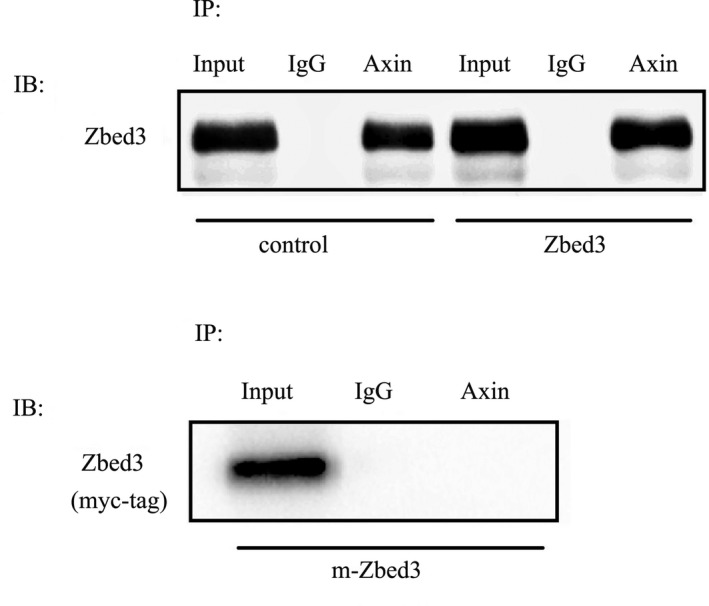
Coimmunoprecipitation of Axin and Zbed3. In A549 cells, Axin binds to wild‐type Zbed3 but not a Zbed3 mutant lacking the Axin binding site

We prepared a mutant Zbed3 plasmid that lacked the Axin binding site. Transfection of wild‐type Zbed3 into A549 and NCI‐H1299 cells significantly up‐regulated β‐catenin expression, whereas transfection of the Zbed3 mutant failed to affect β‐catenin expression (Figure 3). Transfection of wild‐type Zbed3 also significantly up‐regulated p120ctn‐1 expression, whereas this ability was also significantly inhibited when transfected with mutant Zbed3 in cancer cells (Figure 3). Coimmunoprecipitation assays confirmed that Axin (Axin1) bound wild‐type Zbed3 but not the Zbed3 mutant in A549 cells (Figure 4). These results indicate that Zbed3's ability to increase β‐catenin levels is dependent on its binding to Axin.

### 3.3 Zbed3 mutation suppresses its enhancement of cancer cell proliferation and invasiveness

**Figure 5:**
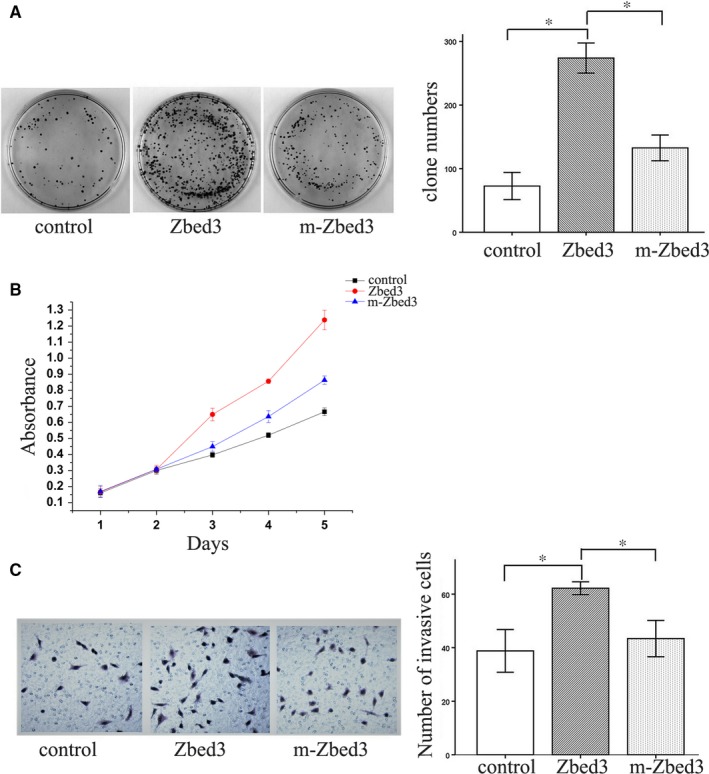
Mutation of the Axin binding site suppresses Zbed3's ability to enhance cancer cell proliferation and invasiveness. Colony formation (A) and MTT (B) assays show that transfection of wild‐type Zbed3 promotes cancer cell proliferation, but this effect is suppressed by transfection of a Zbed3 mutant lacking the Axin binding site (A, B). Transwell assays show that transfection with wild‐type Zbed3 promotes cancer cell invasion, but this action is suppressed by transfection of the Zbed3 mutant (C). The experiments were repeated three times (*P < 0.05)

**Figure 6:**
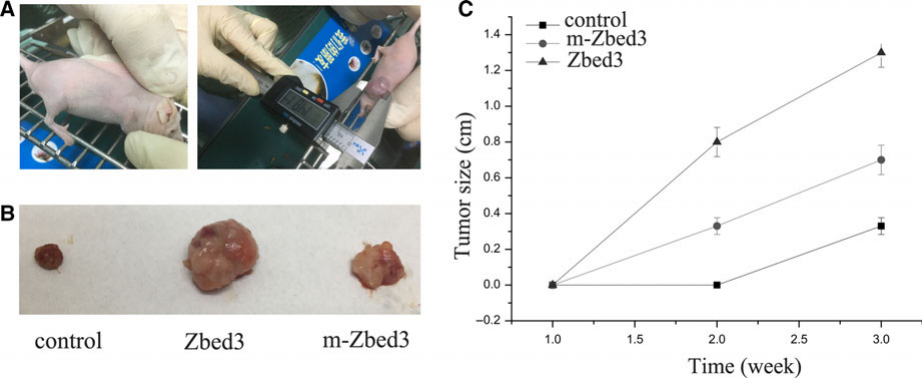
Mutation of the Axin binding site suppresses Zbed3's ability to enhance tumour formation in vivo. Nude mice were injected subcutaneously with tumour cells and tumour formation was examined each week (A). The size of subcutaneously injected tumours were increased in the wild‐type Zbed3 group as compared with the control group, but the effect was significantly inhibited by transfection of the Zbed3 mutant (B, C)

Colony formation and MTT assays showed that transfecting lung cancer cells with wild‐type Zbed3 significantly enhanced their proliferation, but that effect was largely inhibited by transfection of the Zbed3 mutant lacking an Axin binding sited (Figure 5A and B). In addition, Transwell assays showed that transfection of wild‐type Zbed3 significantly increased the invasiveness of cancer cells, but again that effect was largely inhibited transfection of the Zbed3 mutant (Figure 5C). Given that overexpression of the Zbed3 mutant promoted cancer cell proliferation and invasiveness to a small degree (Figure 5), we suggest Zbed3‐mediated enhancement of cancer cell proliferation and invasiveness is in large part, but not entirely, dependent on the function of Axin. To investigate tumour formation in vivo, we used nude mice. The axilla of the mice was injected subcutaneously with tumour cells and tumour formation was examined each week (Figure 6A). In vivo study shows that the size of subcutaneously injected tumours were increased in the wild‐type Zbed3 group as compared with the control group, but the effect was largely inhibited by transfection of the Zbed3 mutant (Figure 6B and C).

### 3.4 Axin knockdown or treatment with a GSK‐3β inhibitor abolished Zbed3‐induced β‐catenin expression in lung cancer cells

**Figure 7:**
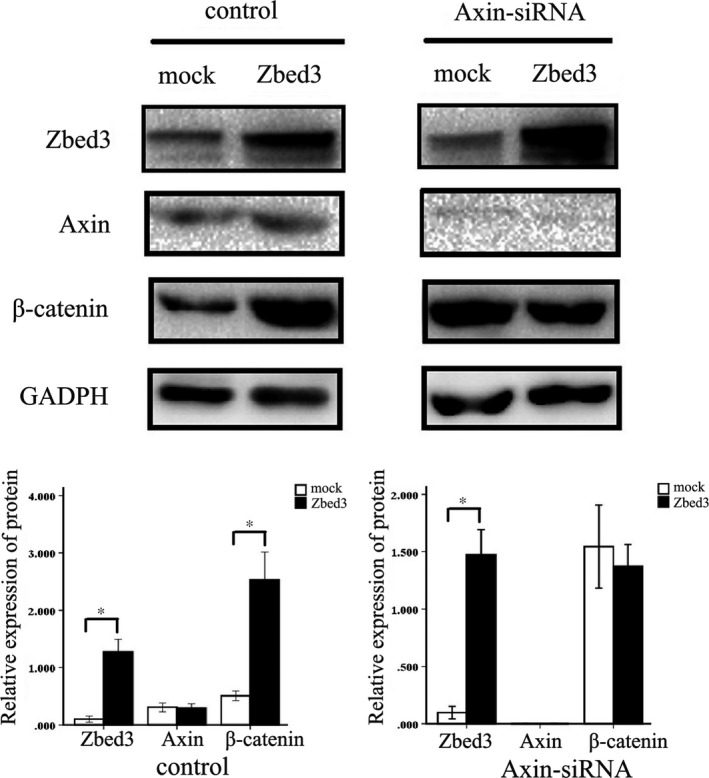
Axin knockdown abolished Zbed3's ability to increase β‐catenin expression in lung cancer cells. Zbed3 overexpression in A549 cells up‐regulated β‐catenin expression. Knocking down Axin abolished this effect. The experiments were repeated three times (*P < 0.05)

**Figure 8:**
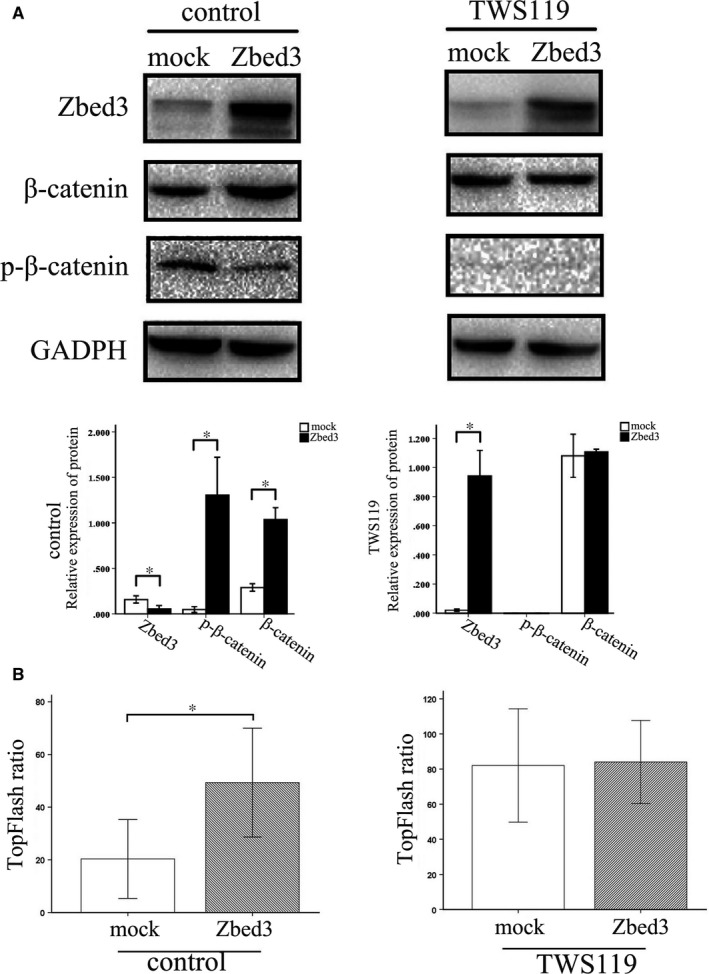
Treatment with a GSK3β inhibitor abolished Zbed3's ability to increase β‐catenin expression and Wnt signalling activity. Western blots show that Zbed3 overexpression increases levels of β‐catenin while decreasing levels of phospho‐β‐catenin in A549 cells, and that this effect is abolished by treating the cells with the GSK3β inhibitor TWS119 (A). Dual‐luciferase assays show that Zbed3 overexpression increases activity in the canonical Wnt signalling pathway in A549 cells, but this effect too was abolished by treatment with TWS119 (B). The experiments were repeated three times (*P < 0.05)

Western blotting showed that Axin is endogenously expressed in A549 cells. Transfection of Zbed3 into A549 cells did not affect Axin expression, but significantly increased β‐catenin expression (Figure 7). However, after Axin knockdown, Zbed3's ability to enhance β‐catenin expression was abolished (Figure 7), indicating the effect was Axin dependent. When we then treated A549 cells with the GSK‐3β inhibitor TWS119, we found that levels of phospho‐β‐catenin were greatly suppressed, and the ability of Zbed3 to increase β‐catenin levels was abolished (Figure 8A). Dual‐luciferase assays showed that activity in the canonical Wnt signalling pathway in A549 cells was significantly increased by Zbed3 transfection. However, the ability of Zbed3 to promote Wnt signalling was abolished in TWS119‐treated cells (Figure 8B). These results indicate that Zbed3's ability to increase β‐catenin expression and Wnt signalling activity was dependent on the activity of the Axin/APC/GSK‐3β complex.

## 4 DISCUSSION

Wnt signalling plays important roles during the development and progression of cancer, including NSCLC.^1^, ^2^, ^3^, ^4^, ^5^, ^6^ We previously observed that Zbed3 is overexpressed in NSCLC tissues compared to normal lung tissues and is associated with cancer development.^7^ Down‐regulation of Zbed3 in lung cancer cells significantly reduced expression of β‐catenin and p120‐catenin 1, both of which are degraded through similar pathways, but the mechanism involved is not completely clear. It is known that β‐catenin degradation is mediated by the Axin/APC/GSK3β complex when Wnt signalling is not activated,^5^, ^6^ and that Zbed3 may regulate β‐catenin by interacting with Axin and inhibiting the function of Axin/APC/GSK3β complex.^10^ Consistent with that finding, abnormal Axin expression or function has been seen in various malignancies, including NSCLC.^8^, ^9^ We therefore investigated the effects of Zbed3 on β‐catenin levels and cancer cell proliferation and invasiveness as it relates to Axin/APC/Gsk3β complex function.

Here, we found that Wnt signalling molecules, including Axin, GSK3β, and β‐catenin, are all expressed in both A549 and NCI‐H1299 cells. Overexpression of Zbed3 significantly increased β‐catenin expression and Wnt signalling in these cancer cells, as well as their proliferation and invasiveness. This is consistent with our previous findings in cancer tissues, which showed that Zbed3 expression was associated with advanced TNM stages and lymph node metastasis of NSCLC.^7^ To determine whether Zbed3 increases β‐catenin through interaction with Axin, we prepared a Zbed3 mutant that lacked the Axin binding site. The results showed that the Zbed3 mutant failed to up‐regulate β‐catenin, which indicates that the interaction with Axin is necessary for Zbed3 to up‐regulate β‐catenin. We also found that overexpression of Zbed3 significantly down‐regulated phospho‐β‐catenin, which indicates Zbed3 may increase β‐catenin levels by inhibiting its degradation mediated via the Axin/APC/GSK3β complex. Knocking down Axin or treating A549 cells with a GSK‐3β inhibitor abolished Zbed3's ability to increase β‐catenin levels and Wnt signalling activity. This suggests that Zbed3 may regulate Wnt signalling in NSCLC cells harbouring the Axin/APC/GSK3β complex by inhibiting the function of this complex. When we transfected cells with a Zbed3 mutant lacking an Axin binding site, we found that the mutant promoted cancer cell proliferation and invasiveness to some degree, but the effect was much smaller than that elicited by wild‐type Zbed3. This indicates that Zbed3's promotion of NSCLC cell proliferation and invasion is largely, but not entirely, through an interaction with Axin. Based on these findings, we suggest that while Zbed3 and Wnt signalling molecules, including Axin, GSK3β, and β‐catenin, are all potential therapeutic targets, there remains a need for further investigation to fully understand the interaction of these molecules in NSCLC.

## CONFLICT OF INTEREST

None.
